# Identification of lurasidone as a potent inhibitor of severe fever with thrombocytopenia syndrome virus by targeting the viral nucleoprotein

**DOI:** 10.3389/fmicb.2025.1578844

**Published:** 2025-04-28

**Authors:** Ting Cheng, Qingcui Xiao, Jing Cui, Shuangjie Dong, Yuqin Wu, Wenqiang Li, Xinya Yang, Lina Ma, Zhiyong Li, Peng Sun, Yinli Xie

**Affiliations:** ^1^School of Basic Medical Sciences, Cixi Biomedical Research Institute, Wenzhou Medical University, Wenzhou, Zhejiang, China; ^2^Institute of Virology, Wenzhou Medical University, Wenzhou, Zhejiang, China; ^3^Institute of Biology, Hebei Academy of Sciences, Shijiazhuang, Hebei, China

**Keywords:** SFTSV, antiviral drugs, molecular docking, nucleoprotein, lurasidone

## Abstract

**Introduction:**

Severe fever with thrombocytopenia syndrome virus (SFTSV) is an emerging tick-borne bunyavirus that causes acute febrile illness with thrombocytopenia and a high mortality rate in humans. Currently, no specific antiviral agents have been approved for the prevention or treatment of SFTSV infection. The viral nucleoprotein (NP) is a critical component involved in viral RNA replication and transcription, representing a promising target for antiviral drug development.

**Methods:**

We performed a structure-based virtual screening of the FDA-approved drug library using AutoDock Vina, aiming to identify potential inhibitors targeting the RNA-binding pocket of SFTSV NP. Promising candidates were further evaluated for antiviral activity in vitro.

**Results:**

Among the screened compounds, lurasidone exhibited strong antiviral activity against SFTSV, with an IC_50_ value of 4.552 μM and a selectivity index (SI) greater than 10, indicating favorable antiviral potency and low cytotoxicity. Mechanistic investigations suggest that lurasidone may exert its inhibitory effect by directly binding to the NP, thereby interfering with viral genome replication.

**Conclusion:**

This study identifies lurasidone as a potential antiviral candidate targeting SFTSV NP and provides a theoretical basis for the repurposing of FDA-approved drugs against emerging viral infections. These findings offer new insights into therapeutic strategies for the treatment of SFTSV.

## 1 Introduction

Severe Fever with Thrombocytopenia Syndrome virus (SFTSV), officially designated as *Dabie bandavirus* or *Bandavirus dabieense*, is a tick-borne virus first isolated from patients with Severe fever with thrombocytopenia syndrome (SFTS) in 2010 (Yu et al., 2011; Liu Q. et al., 2014). The number of SFTS cases has increasing annually, with primary endemic areas located across Asia (Takahashi et al., 2014; Bopp et al., 2020; Huang et al., 2021). The virus poses a serious public health threat, with cases often presenting with symptoms such as high fever and bleeding, and in severe instances, leading to multiple organ failure and death (Ramírez, 2013). SFTSV is primarily transmitted to humans and animals through bites from virus-carrying ticks, particularly *Haemaphysalis longicornis*, which serves as the main vector (Luo et al., 2015; Zhan et al., 2017; Zhuang et al., 2018). Seroprevalence studies have detected SFTSV in various domestic animals, including goats, cattle, dogs, and pigs (Jiao et al., 2012; Niu et al., 2013). These domesticated animals may act as amplifying hosts for SFTSV, contributing significantly to the transmission cycle by supporting tick populations that facilitate viral spread (Jiao et al., 2012; Niu et al., 2013; Zhang et al., 2013). However, despite the growing public health challenge, there are currently no vaccines or specific antiviral treatments available for SFTSV, highlighting the urgent need for further research to develop effective therapeutic strategies.

SFTSV is characterized by a negative-sense, single-stranded RNA genome comprising three segments: large (L), medium (M), and small (S) (Wang et al., 2020). The L segment encodes the RNA-dependent RNA polymerase (RdRp), while the M segment encodes the precursor of the surface glycoproteins Gn and Gc. The S segment employs a bi-cistronic encoding strategy to produce both the nucleoprotein (NP) and non-structural proteins (NSs) (Liu S. et al., 2014). The NP plays a pivotal role in the viral lifecycle by binding to the viral genomic RNA (vRNA) to form ribonucleoprotein complexes (RNPs), which stabilize the vRNA in its polymeric form (Zhou et al., 2013; Lokupathirage et al., 2021). These complexes are crucial for viral replication and packaging (Mo et al., 2020). The RNA-binding cavity, a key structural domain of NP, is critical for SFTSV transcription and replication. Suramin, which occupies the RNA-binding cavity, can effectively inhibit viral infection (Jiao et al., 2013). Therefore, RNA-binding cavity of NP represents a promising target for the development of anti-SFTSV therapeutic strategies.

In recent years, the combined approach of molecular modeling, computational screening, and experimental strategies has gained widespread acceptance in drug repurposing efforts (Onawole et al., 2018; Rosário-Ferreira et al., 2021; Mishra et al., 2024). Computational screening using FDA-approved drug libraries offers a cost-effective and time-efficient approach to identifying potential repurposed drug candidates. Molecular docking is a key technique in computational drug discovery, widely used to study biomolecular interactions and mechanisms and to support structure-based drug design (Forli et al., 2016). Among various docking tools, AutoDock Vina stands out for its speed and accuracy, efficiently predicting noncovalent binding between macromolecules (receptors) and small molecules (ligands) (Trott and Olson, 2010; Forli et al., 2016; Eberhardt et al., 2021).

In this study, we aimed to predict potential drugs targeting the SFTSV NP protein through protein-ligand docking. To reduce low hit rates and false positives often associated with large compound library screenings, we focused on the RNA-binding cavity of NP as the active binding pocket. Using AutoDock Vina molecular docking simulations, we screened potential drug candidates from an FDA-approved drug library. We tested 11 potential drugs currently widely used in clinical practice, covering a range of therapeutic areas, including psychiatric disorders, fungal infections, HCV/HIV infections, acute and chronic diseases, and hematological disorders (Wurzel et al., 2007; Olnes et al., 2012; De Clercq, 2014; Corponi et al., 2019; Fiedorczuk and Chen, 2022). Several compounds demonstrated the ability to inhibit SFTSV infection, with lurasidone emerging as a standout candidate due to its potent antiviral activity. Mechanistic studies suggest that lurasidone may exert its antiviral effects by binding to the viral nucleoprotein, thereby impairing genome replication. These findings provide a valuable theoretical foundation for developing novel antiviral agents to combat SFTSV infection.

## 2 Materials and methods

### 2.1 Cells and virus

Huh-7 cells and Vero cells were cultured in Dulbecco’s modified Eagle’s medium (DMEM) (Cat# 11995500, Gibco) supplemented with 10% heat-inactivated fetal bovine serum (FBS) (Cat# FSP500, ExCell Bio) at 37°C with 5% CO_2_. SFTSV (SDTA-1 strain) was used in the experiments. Virus was passaged in Vero cells. The virus titers were determined by a plaque formation assay.

### 2.2 Antibodies, reagents and compounds

Mouse polyclonal anti-SFTSV nucleoprotein (NP) antibody was kindly provided by Dr. Kaixiao Nie from Shandong First Medical University, and was used to detect viral protein expression in cells via immunofluorescence assays. Goat anti-Mouse IgG (H+L) Alexa Fluor 594 (Cat# A-11005, Thermo Fisher Scientific) was used as a secondary antibody. Dimethyl sulfoxide (DMSO, Cat# D8371) was purchased from Solarbio. Ammonium chloride (Cat# A616422) was purchased from Aladdin. DAPI (Cat# C1006) was obtained from Beyotime. Dihydroergotamine (Cat# HY-B0670A), dutasteride (Cat# HY-13613), daclatasvir (Cat# HY-10466), eltrombopag (Cat# HY-15306), lumacaftor (Cat# HY-13262), glecaprevir (Cat# HY-17634), lurasidone (Cat# HY-B0032A), tolvaptan (Cat# HY-17000), saquinavir (Cat# HY-17007), tadalafil (Cat# HY-90009A), isavuconazonium (Cat# HY-100373) were purchased from MedChemExpress (MCE).

### 2.3 Preparation of protein and ligand structures

The structure of the SFTSV NP pentamer in complex with the small molecule suramin (PDB: 4J4V) was retrieved from the Protein Data Bank (PDB).^1^ Using PyMOL,^2^ all water molecules and the small molecule compound were removed from the structure. The protein structure was then processed using AutoDockTools (ADT, v1.5.7) for hydrogen addition and charge assignment, and saved in PDBQT format. Three-dimensional structures of FDA-approved drug compounds were downloaded from the ZINC15 database in Structure Data File (SDF) format. These molecules were then separated, hydrogenated, and rotatable bonds were defined using OpenBabel (v3.1.1). The processed ligands were saved in PDBQT format to be used as input for docking analysis.

### 2.4 Molecular docking and virtual screening

The binding site for molecular docking was defined based on the position of the ligand suramin in the 4J4V complex. The grid box was centered at the coordinates (67.44, 13.71, 17.20) with a grid size of 28 × 28 × 28 Å. For each ligand docked at the protein binding site, 9 different conformations were generated. AutoDock Vina was employed to conduct the docking process, and molecules with docking scores below −10 kcal/mol were selected for further analysis. The docking procedure was carried out using internally developed scripts. All visualizations were performed using PyMOL (see text footnote 2) and BIOVIA Discovery Studio Visualizer.

### 2.5 RNA extraction and quantitative real-time PCR (qRT-PCR)

Total RNAs were isolated from infected cells with RNeasy Mini Kit (Cat# AP-MN-MS-RNA, Axygen) following the manufacturer’s instructions. Quantitative reverse transcription PCR (qRT-PCR) was carried out with a two-step procedure. First, total RNAs were reverse-transcribed into cDNA with the HiScript III RT SuperMix kit (Cat# R323-01, Vazyme), and then quantified by using Hieff^®^ qPCR SYBR Green Master Mix (Cat# 11201ES03, Yeasen). In brief, each reaction consisted of a total volume of 20 μl containing 0.4 μl of each designed primer (10 μM), 10 μl of Hieff^®^ qPCR SYBR Green Master Mix, 1 μl of cDNA, and 8.2 μl of RNase-free water. qRT–PCR was performed on a Bio-Rad CFX96 Touch Real-Time Detection System. The thermal cycler conditions were set as follows: initial denaturation at 95°C for 5 min, followed by 40 cycles of 95°C for 10 s, 60°C for 30 s. Melting curve analysis was subsequently performed at temperature from 65°C to 95°C to verify the assay specificity. The qRT–PCR primers used are listed as follows.

SFTSV-F sense: 5′-CTGGGCAATGGAAACCGGAAG-3′;
SFTSV-R anti-sense: 5′-CAATGAGGAAGAAGTGAAC AAGT-3′;
GAPDH-F sense: 5′-CAAGAAGGTGGTGAAGCA-3′;
GAPDH-R anti-sense: 5′-AAGGTGGAAGAGTGGGTG-3′.

### 2.6 Cytotoxicity assay

The cytotoxicity of the drugs was assessed using the Cell Counting Kit-8 (CCK-8) assay (Cat# HY-K0301, MCE) on Vero cells. Briefly, 10,000 cells/well were seeded overnight in a 96-well plate. The following day, Vero cells were treated with each drug at concentrations of 5, 10, 20, 40, and 80 μM. An equal concentration of DMSO was incubated as a vehicle control. The cells were then incubated for 48 h (37°C, 5% CO_2_). After incubation, the culture medium was removed, and the cells were washed with phosphate-buffered saline (PBS) (Cat# PB180327, Procell). Next, 100 μl of fresh medium was added to each well, followed by the addition of 10 μl of CCK-8 reagent. The cells were incubated at 37°C for 2 h. The absorbance at 450 nm was measured using the Thermo Scientific Varioskan LUX multifunctional microplate reader. Cell viability was expressed as a percentage of the compound-treated cells relative to the control cells treated with the same concentration of DMSO. The CC_50_ value, representing the cytotoxic concentration at which 50% of the cells remain viable, was calculated.

### 2.7 Calculation of IC_50_

Vero cells were infected with SFTSV at a multiplicity of infection (MOI) of 0.1, with different concentrations of compounds added to the virus dilution and an equal concentration of DMSO was incubated as a vehicle control. After a 1-h incubation at 37°C, the culture medium was discarded, and the cells were washed three times with PBS. Subsequently, the cells were cultured in DMEM medium supplemented with 2% FBS and containing drugs at various concentrations for 48 h. Immunofluorescence analysis (IFA) was performed using an anti-nucleoprotein (NP) antibody (1:400) and Goat anti-Mouse IgG (H+L) Alexa Fluor 594 secondary antibody (1:800) to detect the infected cells. DAPI was used to stain the total number of cells. The percentage of infected cells was quantified using the ImageXpress Micro Confocal System’s analysis tool. Data were fitted to a sigmoid dose-response curve using Prism GraphPad 9.0. The half-maximal inhibitory concentration (IC_50_), representing the concentration of each compound at which 50% of the infection is inhibited, was calculated from the curve.

### 2.8 Immunofluorescence assay (IFA)

Huh-7 and Vero cells were infected with SFTSV simultaneously with drug treatment for a duration of 48 h. An equal concentration of DMSO was incubated as a vehicle control. The cells were washed three times in phosphate-buffered saline (PBS) and fixed with ice-cold methanol (Cat# 1280100101601, Xilong scientific) (−20°C) for 15 min. The cells were then permeabilized with 0.3% Triton X-100 (Cat# BS084, Biosharp) for 20 min, washed with PBS, and blocked in 2% BSA (Cat# 9048-46-8, Solarbio) for 1 h. The cells were subsequently stained with anti-nucleoprotein (NP) antibody (1:400) at 4°C. After washing, the cells were stained with Goat anti-Mouse IgG (H+L) Alexa Fluor 594 secondary antibody (1:800) for 1 h at room temperature. The nuclei were stained with DAPI. Images were examined using a fluorescence microscope Zeiss Axio Observer and the ImageXpress Micro Confocal System (Molecular Devices) to analyze the infection.

### 2.9 Plaque formation assay

Vero cells were seeded in 6-well plates and cultured to 100% confluence. SFTSV was serially diluted from 10^2^ to 10^6^ and used to infect the cells for 1 h. After infection, the medium was removed, and the cells were washed three times with PBS. Fresh medium containing 5% low melting point agarose (Cat# A600015, Sangon Biotech) and 2% FBS in DMEM (2.5 ml per well) was added and allowed to solidify at room temperature for 20 min. The plates were then transferred to a 37°C incubator with 5% CO2 for incubation. On day 6 post-infection, cells were fixed with 4% Paraformaldehyde (PFA) (Cat# G1101, Servicebio) for 2 h. After removing the fixing solution, the agarose gel was rinsed off with running water. The cells were stained with 1% crystal violet solution (Cat# C0121, Beyotime) on a shaker for 15 min. The staining solution was then rinsed off, and the plates were air-dried before counting the number of plaques.

### 2.10 SFTSV binding and internalization assays

For the analysis of viral binding, Vero cells were pretreated with lurasidone or DMSO for 1 h at 37°C. The cells were then transferred to ice and infected with a mixture of the drug and virus at a MOI of 5 for 1 h at 4°C. An equal concentration of DMSO was incubated as a vehicle control. After incubation, the inoculum was removed, and the cells were washed three times with pre-chilled PBS. The relative levels of bound viral particles were quantified by qRT-PCR, as described above. For the analysis of viral internalization, Vero cells were pretreated with lurasidone or DMSO at 37°C for 1 h. The cells were then transferred to ice and infected with the virus-drug mixture at a MOI of 5 for 1 h at 4°C. After removing the inoculum, the cells were washed three times with pre-chilled PBS, and 20 mM ammonium chloride was added to prevent viral fusion. The cells were incubated at 37°C for 2 h to allow for internalization. Following incubation, the cells were washed with PBS and treated with trypsin to remove any surface-bound viral particles. The internalized viral particles were quantified by qRT-PCR as described previously.

### 2.11 SFTSV post-entry assay

Vero cells were seeded into 24-well plates at a density of 1 × 10^5^ cells per well. After 12 h, the cells were incubated with SFTSV at 37°C for 1 h to allow virus entry. The medium was then replaced, and the cells were treated with lurasidone at 37°C for 48 h. An equal concentration of DMSO was incubated as a vehicle control. After 48 h of infection, viral RNA levels were quantified using qRT-PCR, and the viral titer in the supernatant was measured using the plaque formation assay.

### 2.12 Quantification and statistical analysis

All analyses were performed with GraphPad Prism statistical software. The data are expressed as the means ± SEMs and were statistically analyzed with a two-tailed unpaired Student’s *t*-test. A *P*-value of <0.05 was considered to indicate statistical significance. *P < 0.05, **P < 0.01, ***P < 0.001, ns, not significant (*P* ≥ 0.05).

## 3 Results

### 3.1 Virtual screening of potential inhibitors targeting SFTSV NP

**FIGURE 1:**
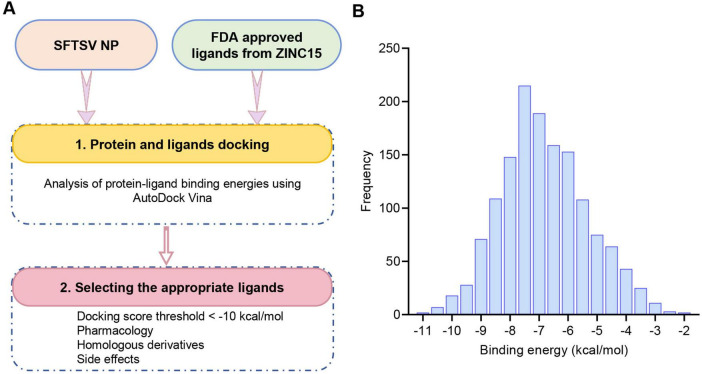
Structure-based virtual screening of the FDA drug database for anti-SFTSV NP molecular docking. **(A)** Workflow diagram of the virtual screening cascade protocol. **(B)** Results of the virtual screening of the FDA drug library using AutoDock Vina targeting the RNA-binding domain of SFTSV NP. The bar graph shows the number of compounds with predicted binding free energies within intervals of 0.5 kcal/mol bins.

The RNA-binding cavity of the SFTSV NP is essential for viral transcription and replication (Sun et al., 2018). Small molecules that occupy this cavity can effectively inhibit viral infection by disrupting the interaction between NP and the viral RNA. In this study, a total of 1430 FDA-approved drugs from the ZINC15 database were docked to the prepared protein receptor as described above. AutoDock Vina was employed as docking software. An in-house bash script was used to execute the docking program to screen multiple molecules (Figure 1A). The list of these compounds, along with their database IDs and calculated binding free energy (ΔG), is provided in Supplementary Table 1. As shown in Figure 1B, the docking scores of the total hits against NP ranged from −11.5 to −2.5 kcal/mol. From these compounds, the top 27 molecules were selected based on docking scores below −10 kcal/mol. These candidates are considered to have favorable interactions with SFTSV NP. Subsequently, these candidates were further filtered based on factors such as chemical structure similarity, pharmacological profiles, cytotoxicity, solubility, and potential side effects. Ultimately, 11 FDA-approved drugs were selected for experimental validation. These 11 drugs are used for a range of conditions, including viral infections (HCV/HIV), fungal infections, chronic diseases like cystic fibrosis, benign prostatic hyperplasia, and thrombocytopaenia. Additionally, they treat acute conditions such as migraines, erectile dysfunction, and pulmonary hypertension (Table 1).

**TABLE 1:** Molecular docking of selected FDA approved drugs against the nucleoproteins of SFTSV.

Name	Docking score (kcal/mol)	Target	Treatment
Dihydroergotamine	−11	Agonist of 5-hydroxytryptamine (5HT) receptors	Migraines
Dutasteride	−10.8	5-alpha reductase inhibitor	Benign prostatic hyperplasia
Daclatasvir	−10.7	Hepatitis C Virus (HCV) NS5A inhibitor	HCV infection
Eltrombopag	−10.3	Thrombopoietin receptor agonist	Thrombocytopenia and aplastic anemia
Lumacaftor	−10.3	Stabilizes mutated cystic fibrosis transmembrane conductance regulator (CFTR) conformation	Cystic fibrosis
Glecaprevir	−10.3	HCV NS3/4A protease inhibitor	HCV infection
Lurasidone	−10.2	Dopamine D2 and serotonin 5-HT1 receptor antagonist	Schizophrenia and bipolar depression
Tolvaptan	−10.1	Vasopressin V2-receptor antagonist	Autosomal dominant polycystic kidney disease
Saquinavir	−10.1	HIV-1 protease inhibitor	HIV infection
Tadalafil	−10.1	Phosphodiesterase-5 inhibitor	Erectile dysfunction and pulmonary arterial hypertension
Isavuconazonium	−10.1	Lanosterol 14-alpha demethylase inhibitor	Invasive aspergillosis and mucormycosis

### 3.2 In vitro characterization of anti-SFTSV drug candidates

**FIGURE 2:**
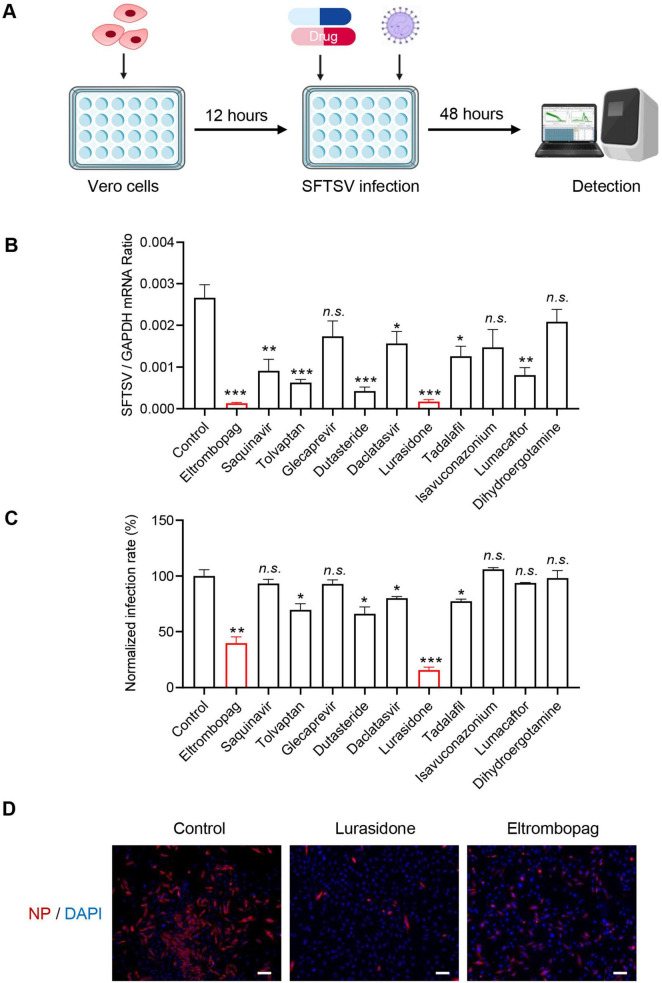
Identification antiviral compounds against SFTSV. **(A)** Schematic representation of the study design. **(B)** Compounds (10 μM) were mixed with SFTSV [0.1 multiplicity of infection (MOI)] to infect Vero cells. DMSO was used as a control. Infected cells were collected 48 h post-infection (hpi), and viral genomes were detected by qRT-PCR. **(C)** Cell-based high-throughput screening to identify inhibitors of SFTSV infection. Compounds (10 μM) were mixed with SFTSV (MOI = 0.1) to infect Vero cells. DMSO was used as a control. After 48 h of SFTSV infection, cells were stained with anti-NP, and nucleus were stained with DAPI. Images were acquired using the ImageXpress Micro Confocal System (Molecular Devices) to analyze the infection. The infection rate of DMSO control was set as 100%, and the infection rate of each drug was calculated by normalizing to DMSO control. **(D)** Lurasidone (10 μM) and eltrombopag (10 μM) were each mixed with SFTSV (MOI = 0.1) to infect Huh-7 cells. After 48 h of SFTSV infection. Cells were stained with anti-NP, and nucleus were stained with DAPI. Images were acquired by Zeiss Axio Observer microscopy. Scale bars:100 μm. Statistical significance: *p < 0.05, **p < 0.01, ***p < 0.001.

Following the virtual screening, further *in vitro* experiments were conducted to validate the antiviral effects of the identified drugs. Vero cells were infected with SFTSV and treated with candidate compounds or the vehicle control (DMSO) for 48 h. The intracellular viral RNA levels were quantified using qRT-PCR (Figure 2A). Among eight clinically approved drugs (eltrombopag, saquinavir, tolvaptan, dutasteride, daclatasvir, lurasidone, tadalafil and lumacaftor) exhibiting potent antiviral activity, eltrombopag and lurasidone demonstrated the most potent antiviral activity, achieving > 10-fold reduction in viral RNA levels compared to the control group (Figure 2B). Subsequently, immunofluorescence analysis was performed to assess the inhibitory effects of the drugs on infection rates. Consistent with the qRT-PCR results, lurasidone and eltrombopag significantly reduced the SFTSV infection rate in Vero cells (Figure 2C). Furthermore, the antiviral effects of lurasidone and eltrombopag were confirmed in Huh-7 cells, consistent with the results observed in Vero cells, showing a significant reduction in the number of infected cells (Figure 2D). These results suggest that both lurasidone and eltrombopag are promising candidates for combating SFTSV infections.

### 3.3 Acute cytotoxicity and antiviral dose-response analysis of the drug candidates

**FIGURE 3:**
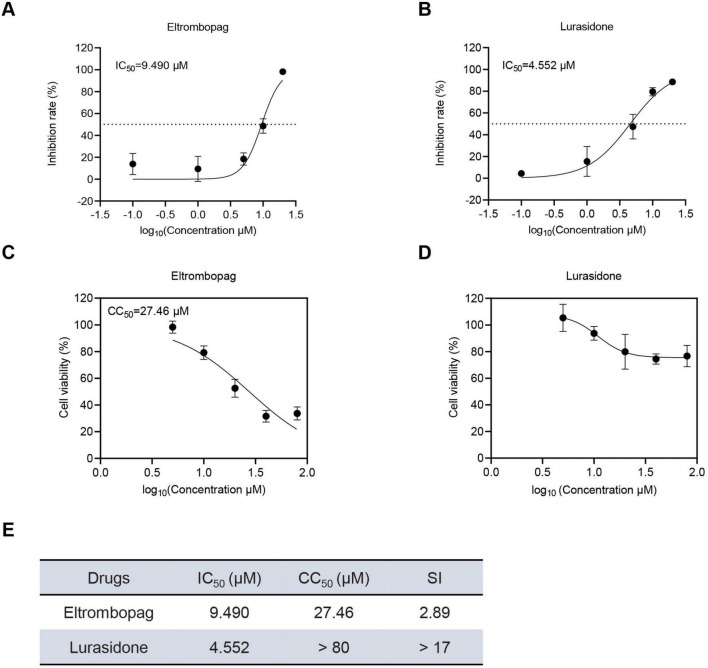
Dose-dependent inhibitory effects and cell viability analysis of Lurasidone and Eltrombopag in Vero cells. **(A,B)** Vero cells were treated with each drug at the indicated concentrations and inoculated with SFTSV at a MOI of 0.1. At 48 hpi, cells were fixed, and infected cells were detected by immunofluorescence analysis using antibody against NP. IC_50_: 50% inhibitory concentration. **(C,D)** Vero cells were treated with each drug at the indicated concentrations. At 48 hpi, cell viability was measured using the CCK-8 assay. CC_50_: 50% cytotoxic concentration. **(E)** Dose-dependent inhibitory effects and SI of lurasidone and eltrombopag are summarized.

To further evaluate the antiviral potential of the promising drug candidates, eltrombopag and lurasidone, their cytotoxicity and antiviral efficacy were assessed by determining their half-maximal cytotoxic concentration (CC_50_) and half-maximal inhibitory concentration (IC_50_). As shown in Figures 3A, B, the IC_50_ values for eltrombopag and lurasidone against SFTSV were found to be 9.49 and 4.552 μM, respectively. In parallel, the 50% cytotoxic concentration (CC_50_) of eltrombopag in Vero cells was 27.46 μM, but lurasidone exhibited a CC_50_ greater than 80 μM (Figures 3C, D). Furthermore, the selectivity index (SI) for lurasidone was calculated to be greater than 17 (Figure 3E), significantly higher than the SI for eltrombopag. indicating that lurasidone may be a more promising candidate for antiviral therapy. Next, we evaluated the inhibitory effects of lurasidone on SFTSV replication at different time points. The results demonstrated that lurasidone significantly suppressed SFTSV replication at both 48 and 72 h post-infection (Supplementary Figure 1). These results underscore lurasidone’s potential as a candidate for further development in the treatment of SFTSV infections, offering superior antiviral efficacy and a better safety profile compared to eltrombopag.

### 3.4 Mechanistic investigation of Lurasidone’s antiviral activity

**FIGURE 4:**
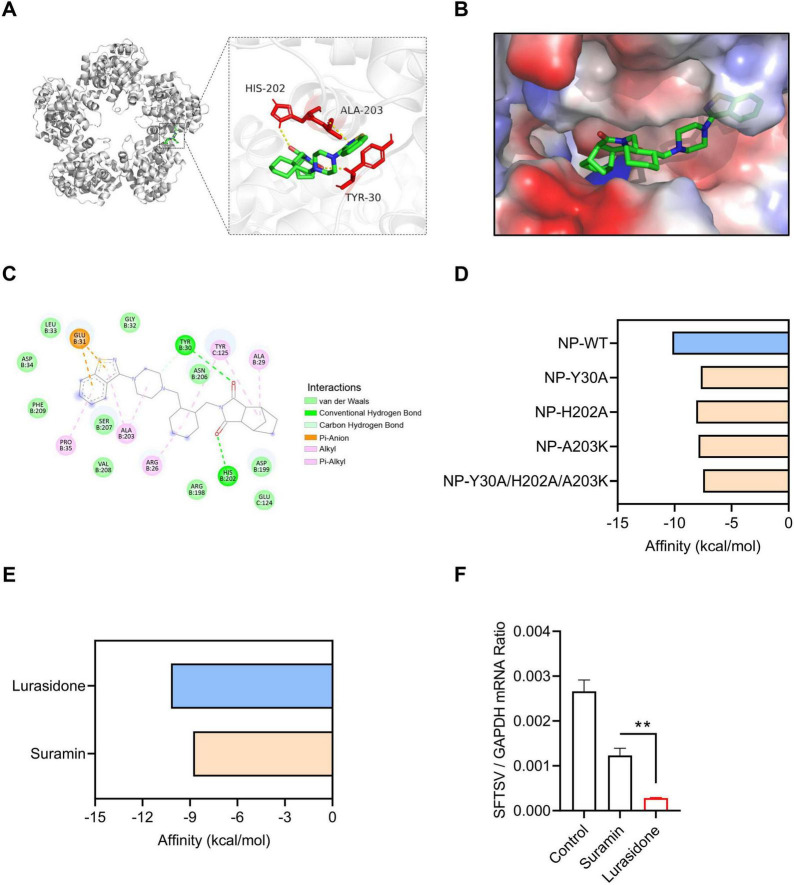
Molecular docking interactions of Lurasidone with SFTSV NP. **(A)** Molecular docking analysis predicts the amino acid residues at the interaction interface between NP and lurasidone. **(B)** Close-up view of the interaction interface between NP and lurasidone, showing the protein surface. **(C)** A 2D schematic representation of the interaction profile between NP and lurasidone. **(D)** ΔG calculation for mutants of NP (Y30A, H202A, A203K) docked with lurasidone by AutoDock Vina. **(E)** ΔG calculation for NP docked with lurasidone and suramin by AutoDock Vina. **(F)** Lurasidone or suramin (20 μM) were mixed with SFTSV (MOI = 0.1) to infect Vero cells. DMSO was used as a control. Infected cells were collected at 48 h post-infection (hpi), and viral genomes were detected by qRT-PCR. Statistical significance: **p < 0.01.

To further investigate the mechanism by which lurasidone inhibits SFTSV replication, we conducted molecular docking simulations to analyze its interaction with the SFTSV NP. The docking model revealed that lurasidone specifically interacts with the RNA-binding cavity of NP, located in a deep cavity formed at the interface of the protein pentamer (Figures 4A, B). The docking results showed that lurasidone forms stable interactions with NP, primarily through hydrogen bonding and hydrophobic interactions. Specifically, lurasidone forms three hydrogen bonds with residues Tyr 30 and His 202, and four hydrophobic interactions with Ala 203, Glu 31, and Tyr 125 (Figure 4C). The binding free energy of lurasidone with NP was calculated to be −10.2 kcal/mol. Molecular docking simulation results demonstrated that mutation of the interaction sites (Y30A, H202A, A203K) led to an increase in binding free energy, indicating the critical role of these residues in maintaining the interaction between lurasidone and NP (Figure 4D). Next, we analyzed the binding profiles of lurasidone and suramin with NP. Although both lurasidone and suramin bind to the RNA-binding cavity of SFTSV NP, docking results revealed that they occupy different spatial sites within the cavity (Supplementary Figure 2). The binding free energy of lurasidone with NP is lower than that of suramin, suggesting a stronger binding affinity of lurasidone for SFTSV NP (Figure 4E). The results of qRT-PCR showed that both suramin and lurasidone significantly reduced the viral RNA levels in SFTSV-infected cells (Figure 4F), with lurasidone demonstrating a more pronounced inhibitory effect. Furthermore, we analyzed the binding free energy of lurasidone with various human RNA-binding proteins and other viral RNA-binding proteins. The results showed that lurasidone exhibited a significantly lower binding free energy with SFTSV NP compared to other RNA-binding proteins (Supplementary Figure 3), suggesting a high specificity for NP. These findings suggest that lurasidone potentially binds more stably to NP, thereby disrupting its role in the viral replication cycle.

### 3.5 Mechanistic analysis of Lurasidone’s post-entry inhibition

**FIGURE 5:**
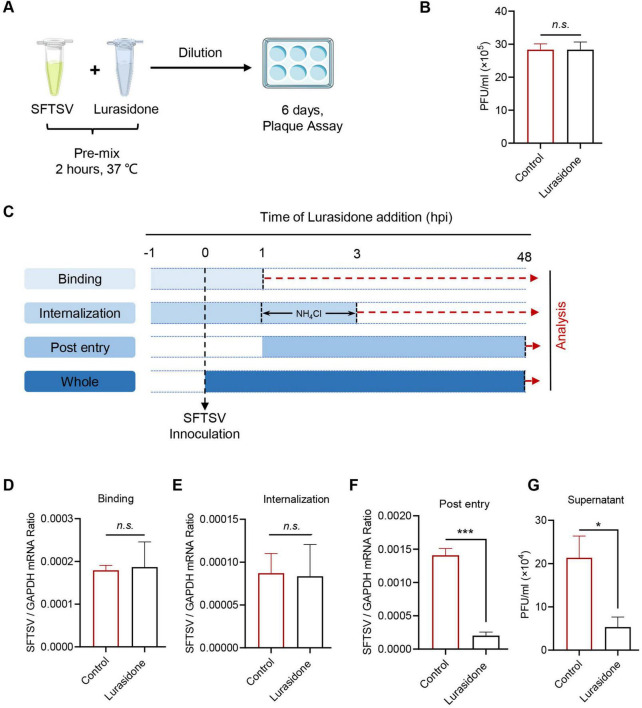
Antiviral mechanism of action of Lurasidone. **(A,B)** SFTSV inactivation assay. SFTSV were mixed with lurasidone (10 μM) and incubated for 2 h, followed by standard plaque assay from diluting the mixture. **(C)** Schematic of the time-of-addition analysis to examine the steps in the SFTSV life cycle. Lurasidone was added at different time points during virus infection. For binding analysis, cells were pretreated with lurasidone for 1 h, followed by SFTSV infection at 4°C for 1 h, and unbound virus was removed. For internalization analysis, cells were pretreated with lurasidone for 1 h, infected with SFTSV at 4°C for 1 h, then shifted to 37°C for 2 h with ammonium chloride to block viral fusion, and unbound virus was removed. For post-entry analysis, lurasidone was added after viral binding and entry, and remained for the subsequent 48 h of infection. **(D–F)** Inhibitory effects of lurasidone under different conditions were assessed by measuring viral genomes by qRT-PCR. **(G)** Viral titers during the post-entry stage were determined by a plaque-forming assay. Statistical significance: *p < 0.05, ***p < 0.001.

To further elucidate the mechanism by which lurasidone inhibits SFTSV replication, we first tested whether lurasidone could directly disrupt the infectivity of viral particles. Lurasidone was incubated with SFTSV *in vitro* for 2 h, followed by dilution and analysis of viral infectivity using a plaque formation assay. The results showed that *in vitro* incubation with lurasidone did not inhibit SFTSV infectivity in Vero cells (Figures 5A, B), indicating that lurasidone does not directly inactivate the virus. Given that viral infection of host cells involves multiple stages, we next assessed whether lurasidone affects virus binding, internalization, or post-entry processes (Shen et al., 2022). For binding analysis, cells were pretreated with lurasidone for 1 h, followed by infection with SFTSV at 4°C for 1 h, after which unbound virus was removed. For internalization analysis, cells were pretreated with lurasidone for 1 h, followed by infection with SFTSV at 4°C for 1 h, following which the cell culture supernatant was replaced with a fresh medium containing ammonium chloride to block viral fusion with the plasma membrane from 4°C to 37°C for 2 h. Then any virus bound to the cell surface was removed. For post-entry analysis, lurasidone was added after the viral binding and entry phases and remained present for the remaining 48 h of infection (Figure 5C). Viral RNA levels were measured to evaluate the inhibitory impact of lurasidone. The results of qRT-PCR showed lurasidone had not significantly decreased viral RNA level during binding or internalization stages (Figures 5D, E), while lurasidone significantly attenuate viral RNA levels during the stage of post-entry (Figure 5F). The results indicated that lurasidone did not affect the binding or internalization stages of SFTSV infection but significantly inhibited the post-entry phase. Furthermore, lurasidone treatment during the post-entry phase reduced the production of infectious viral particles (Figure 5G). These findings signify that lurasidone predominantly hampers intracellular viral replication.

## 4 Discussion

Virtual screening, leveraging molecular docking simulations to identify potential antiviral candidates, has emerged as a powerful tool for rapid drug discovery (Kumar et al., 2022). For example, DW-D-5, a novel HIV inhibitor, was identified through docking screening of the Maybridge database targeting the p75 binding site of HIV-1 integrase. Notably, combining DW-D-5 with FDA-approved anti-HIV drugs demonstrated additive inhibitory effects on HIV-1 replication, highlighting its potential for combination therapy (Wang et al., 2017). Similarly, computational modeling and experimental validation of 15,220 commercially available small molecules led to the identification of corilagin (RAI-S-37) as a non-nucleoside inhibitor of SARS-CoV-2 RdRp. Corilagin effectively inhibited polymerase activity and potently suppressed viral infection (Li et al., 2021). Additionally, virtual screening of over 527,000 natural compounds using Autodock Vina revealed an active compound as a novel HSV-1 inhibitor (Wu et al., 2022). In this study, we used AutoDock Vina to perform virtual screening, as it is one of the most popular molecular docking tools due to its high docking speed and accuracy. AutoDock Vina employs a scoring function to predict the binding affinity between small molecules and protein targets, making it an efficient tool for large-scale virtual screening and drug discovery (Biesiada et al., 2011; Suhandi et al., 2024). We screened FDA-approved drugs to identify potential candidates for inhibiting SFTSV infection. A total of 11 potential drugs currently used in clinical practice were tested, covering a wide range of therapeutic areas, including psychiatric disorders (Orzelska-Górka et al., 2022), fungal infections (McCormack, 2015), HCV/HIV infections (Figgitt and Plosker, 2000; Smolders et al., 2019), and hematological disorders (Townsley et al., 2017). Among these, lurasidone was identified as an effective inhibitor of SFTSV infection *in vitro*. Since lurasidone has already been approved by the FDA, this approach circumvents the lengthy and expensive preclinical safety testing, thereby accelerating the development of antiviral therapies (Li et al., 2023).

Previous studies have identified several effective antiviral agents against SFTSV. Ribavirin, a nucleotide analog with broad-spectrum antiviral activity against various viruses, effectively blocks SFTSV replication *in vitro* (Tani et al., 2016; Lee et al., 2017). However, clinical studies have shown no difference in the case fatality rate between patients treated with ribavirin and those who did not receive the drug (Lu et al., 2015). Calcium channel blockers (CCBs) have also demonstrated the ability to inhibit SFTSV replication *in vitro* by impairing virus internalization and genome replication (Li et al., 2019). Furthermore, recent findings have shown that elevated levels of the secondary bile acid taurolithocholic acid (TLCA) are associated with reduced fatality rates and suppressed viraemia in SFTSV-infected patients. Treatment with TLCA has also been shown to protect mice from lethal SFTSV infection (Zheng et al., 2024). Another promising candidate, suramin, has been shown to bind to the putative RNA-binding cavity of the SFTSV nucleoprotein (NP), effectively inhibiting SFTSV replication (Jiao et al., 2013). In this study, we found that lurasidone also targets the RNA-binding cavity of SFTSV NP to inhibit infection. Although both lurasidone and suramin bind to the RNA-binding cavity of SFTSV NP, docking results revealed that they occupy different spatial sites within the cavity. Lurasidone exhibits a lower binding free energy of −10.2 kcal/mol, indicating stronger binding affinity. Experimental results further confirmed that lurasidone demonstrates superior inhibitory effects on NP compared to suramin.

Lurasidone is a second-generation antipsychotic classified as an atypical antipsychotic, primarily used in the treatment of schizophrenia and bipolar disorder (Miura et al., 2023). It has been approved for clinical use in many countries worldwide and is associated with fewer side effects compared to other antipsychotic medications, particularly in terms of weight gain and metabolic disturbances (Fiorillo et al., 2022; Miura et al., 2023). Recent studies have also indicated that lurasidone can inhibit SARS-CoV-2 and the human coronavirus HCoV-OC43 (Milani et al., 2021), suggesting its potential as an antiviral agent. Additionally, computational studies have proposed that lurasidone may serve as an inhibitor of the H7N9 neuraminidase protein (Mtambo and Kumalo, 2022). These findings underscore lurasidone’s promise as a therapeutic candidate beyond its current psychiatric applications, opening new avenues for the treatment of various viral infections.

In summary, our research highlights lurasidone’s potential as an antiviral agent against SFTSV, demonstrating its ability to inhibit replication by binding to the NP protein. Lurasidone primarily acts during the post-entry phase, reducing viral replication and the production of infectious particles. Due to the rapid mutation rate of RNA viruses, antiviral therapies targeting viral proteins often lead to the development of drug-resistant mutants (Lingappa et al., 2013; Mottram et al., 2017). To assess the potential for lurasidone-induced resistance, we conducted serial passaging of SFTSV in the presence of lurasidone. Interestingly, no resistant variants emerged after five passages (data not shown). Further passaging may be necessary to fully evaluate the risk of resistance development. These findings position lurasidone as a promising candidate for antiviral development. Additionally, drug repurposing and virtual screening were key strategies in identifying effective treatments for emerging viral diseases. Our work contributes to the growing body of knowledge on antiviral drug development and underscores the importance of innovative approaches like drug repurposing and computational screening in addressing global health challenges.

## Data Availability

The datasets presented in this study can be found in online repositories. The names of the repository/repositories and accession number(s) can be found in this article/Supplementary material.

## Footnotes

1
https://www.rcsb.org/search

2
https://pymol.org

## References

[B1] Biesiada J. Porollo A. Velayutham P. Kouril M. Meller J. (2011). Survey of public domain software for docking simulations and virtual screening. *Hum. Genomics* 5 497–505. 10.1186/1479-7364-5-5-497 21807604 PMC3525969

[B2] Bopp N. E. Kaiser J. A. Strother A. E. Barrett A. D. T. Beasley D. W. C. Benassi V. (2020). Baseline mapping of severe fever with thrombocytopenia syndrome virology, epidemiology and vaccine research and development. *NPJ Vacc.* 5:111. 10.1038/s41541-020-00257-5 33335100 PMC7746727

[B3] Corponi F. Fabbri C. Bitter I. Montgomery S. Vieta E. Kasper S. (2019). Novel antipsychotics specificity profile: A clinically oriented review of lurasidone, brexpiprazole, cariprazine and lumateperone. *Eur. Neuropsychopharmacol.* 29 971–985. 10.1016/j.euroneuro.2019.06.008 31255396

[B4] De Clercq E. (2014). Current race in the development of DAAs (direct-acting antivirals) against HCV. *Biochem. Pharmacol.* 89 441–452. 10.1016/j.bcp.2014.04.005 24735613

[B5] Eberhardt J. Santos-Martins D. Tillack A. F. Forli S. (2021). AutoDock Vina 1.2.0: New docking methods, expanded force field, and python bindings. *J. Chem. Inf. Model* 61 3891–3898. 10.1021/acs.jcim.1c00203 34278794 PMC10683950

[B6] Fiedorczuk K. Chen J. (2022). Mechanism of CFTR correction by type I folding correctors. *Cell* 185:158–168.e111. 10.1016/j.cell.2021.12.009. 34995514

[B7] Figgitt D. P. Plosker G. L. (2000). Saquinavir soft-gel capsule: An updated review of its use in the management of HIV infection. *Drugs* 60 481–516. 10.2165/00003495-200060020-00016 10983742

[B8] Fiorillo A. Cuomo A. Sampogna G. Albert U. Calò P. Cerveri G. (2022). Lurasidone in adolescents and adults with schizophrenia: From clinical trials to real-world clinical practice. *Expert Opin. Pharmacother.* 23 1801–1818. 10.1080/14656566.2022.2141568 36398838

[B9] Forli S. Huey R. Pique M. E. Sanner M. F. Goodsell D. S. Olson A. J. (2016). Computational protein-ligand docking and virtual drug screening with the AutoDock suite. *Nat. Protoc.* 11 905–919. 10.1038/nprot.2016.051 27077332 PMC4868550

[B10] Huang X. Li J. Li A. Wang S. Li D. (2021). Epidemiological characteristics of severe fever with thrombocytopenia syndrome from 2010 to 2019 in Mainland China. *Int. J. Environ. Res. Public Health* 18:3092. 10.3390/ijerph18063092 33802869 PMC8002760

[B11] Jiao L. Ouyang S. Liang M. Niu F. Shaw N. Wu W. (2013). Structure of severe fever with thrombocytopenia syndrome virus nucleocapsid protein in complex with suramin reveals therapeutic potential. *J. Virol.* 87 6829–6839. 10.1128/jvi.00672-13 23576501 PMC3676114

[B12] Jiao Y. Zeng X. Guo X. Qi X. Zhang X. Shi Z. (2012). Preparation and evaluation of recombinant severe fever with thrombocytopenia syndrome virus nucleocapsid protein for detection of total antibodies in human and animal sera by double-antigen sandwich enzyme-linked immunosorbent assay. *J. Clin. Microbiol.* 50 372–377. 10.1128/jcm.01319-11 22135253 PMC3264160

[B13] Kumar S. Kovalenko S. Bhardwaj S. Sethi A. Gorobets N. Y. Desenko S. M. (2022). Drug repurposing against SARS-CoV-2 using computational approaches. *Drug Discov. Today* 27 2015–2027. 10.1016/j.drudis.2022.02.004 35151891 PMC8830191

[B14] Lee M. J. Kim K. H. Yi J. Choi S. J. Choe P. G. Park W. B. (2017). In vitro antiviral activity of ribavirin against severe fever with thrombocytopenia syndrome virus. *Korean J. Intern. Med.* 32 731–737. 10.3904/kjim.2016.109 27899013 PMC5511939

[B15] Li G. Hilgenfeld R. Whitley R. De Clercq E. (2023). Therapeutic strategies for COVID-19: Progress and lessons learned. *Nat. Rev. Drug Discov.* 22 449–475. 10.1038/s41573-023-00672-y 37076602 PMC10113999

[B16] Li H. Zhang L. K. Li S. F. Zhang S. F. Wan W. W. Zhang Y. L. (2019). Calcium channel blockers reduce severe fever with thrombocytopenia syndrome virus (SFTSV) related fatality. *Cell Res.* 29 739–753. 10.1038/s41422-019-0214-z 31444469 PMC6796935

[B17] Li Q. Yi D. Lei X. Zhao J. Zhang Y. Cui X. (2021). Corilagin inhibits SARS-CoV-2 replication by targeting viral RNA-dependent RNA polymerase. *Acta Pharm. Sin. B* 11 1555–1567. 10.1016/j.apsb.2021.02.011 33614402 PMC7883726

[B18] Lingappa U. F. Wu X. Macieik A. Yu S. F. Atuegbu A. Corpuz M. (2013). Host-rabies virus protein-protein interactions as druggable antiviral targets. *Proc. Natl. Acad. Sci. U. S. A.* 110 E861–E868. 10.1073/pnas.1210198110 23404707 PMC3593902

[B19] Liu Q. He B. Huang S. Y. Wei F. Zhu X. Q. (2014). Severe fever with thrombocytopenia syndrome, an emerging tick-borne zoonosis. *Lancet Infect. Dis.* 14 763–772. 10.1016/s1473-3099(14)70718-2 24837566

[B20] Liu S. Chai C. Wang C. Amer S. Lv H. He H. (2014). Systematic review of severe fever with thrombocytopenia syndrome: Virology, epidemiology, and clinical characteristics. *Rev. Med. Virol.* 24 90–102. 10.1002/rmv.1776 24310908 PMC4237196

[B21] Lokupathirage S. M. W. Tsuda Y. Ikegame K. Noda K. Muthusinghe D. S. Kozawa F. (2021). Subcellular localization of nucleocapsid protein of SFTSV and its assembly into the ribonucleoprotein complex with L protein and viral RNA. *Sci. Rep.* 11:22977. 10.1038/s41598-021-01985-x 34836987 PMC8626419

[B22] Lu Q. B. Zhang S. Y. Cui N. Hu J. G. Fan Y. D. Guo C. T. (2015). Common adverse events associated with ribavirin therapy for severe fever with thrombocytopenia syndrome. *Antiviral Res.* 119 19–22. 10.1016/j.antiviral.2015.04.006 25892251

[B23] Luo L. M. Zhao L. Wen H. L. Zhang Z. T. Liu J. W. Fang L. Z. (2015). Haemaphysalis longicornis ticks as reservoir and vector of severe fever with thrombocytopenia syndrome virus in China. *Emerg. Infect. Dis.* 21 1770–1776. 10.3201/eid2110.150126 26402039 PMC4593435

[B24] McCormack P. L. (2015). Isavuconazonium: First global approval. *Drugs* 75 817–822. 10.1007/s40265-015-0398-6 25902926

[B25] Milani M. Donalisio M. Bonotto R. M. Schneider E. Arduino I. Boni F. (2021). Combined in silico and in vitro approaches identified the antipsychotic drug lurasidone and the antiviral drug elbasvir as SARS-CoV2 and HCoV-OC43 inhibitors. *Antiviral Res.* 189:105055. 10.1016/j.antiviral.2021.105055 33713730 PMC7944860

[B26] Mishra A. S. Vasanthan M. Malliappan S. P. (2024). Drug repurposing: A leading strategy for new threats and targets. *ACS Pharmacol. Transl. Sci.* 7 915–932. 10.1021/acsptsci.3c00361 38633585 PMC11019736

[B27] Miura I. Horikoshi S. Ichinose M. Suzuki Y. Watanabe K. (2023). Lurasidone for the treatment of schizophrenia: Design, development, and place in therapy. *Drug Des. Devel Ther.* 17 3023–3031. 10.2147/dddt.S366769 37789971 PMC10544203

[B28] Mo Q. Xu Z. Deng F. Wang H. Ning Y. J. (2020). Host restriction of emerging high-pathogenic bunyaviruses via MOV10 by targeting viral nucleoprotein and blocking ribonucleoprotein assembly. *PLoS Pathog.* 16:e1009129. 10.1371/journal.ppat.1009129 33284835 PMC7746268

[B29] Mottram T. J. Li P. Dietrich I. Shi X. Brennan B. Varjak M. (2017). Mutational analysis of Rift Valley fever phlebovirus nucleocapsid protein indicates novel conserved, functional amino acids. *PLoS Negl. Trop. Dis.* 11:e0006155. 10.1371/journal.pntd.0006155 29267287 PMC5764413

[B30] Mtambo S. E. Kumalo H. M. (2022). In silico drug repurposing of FDA-approved drugs highlighting promacta as a potential inhibitor of H7N9 influenza virus. *Molecules* 27:4515. 10.3390/molecules27144515 35889388 PMC9321947

[B31] Niu G. Li J. Liang M. Jiang X. Jiang M. Yin H. (2013). Severe fever with thrombocytopenia syndrome virus among domesticated animals, China. *Emerg. Infect. Dis.* 19 756–763. 10.3201/eid1905.120245 23648209 PMC3647489

[B32] Olnes M. J. Scheinberg P. Calvo K. R. Desmond R. Tang Y. Dumitriu B. (2012). Eltrombopag and improved hematopoiesis in refractory aplastic anemia. *N. Engl. J. Med.* 367 11–19. 10.1056/NEJMoa1200931 22762314 PMC3422737

[B33] Onawole A. T. Kolapo T. U. Sulaiman K. O. Adegoke R. O. (2018). Structure based virtual screening of the Ebola virus trimeric glycoprotein using consensus scoring. *Comput. Biol. Chem.* 72 170–180. 10.1016/j.compbiolchem.2017.11.006 29361403

[B34] Orzelska-Górka J. Mikulska J. Wiszniewska A. Biała G. (2022). New Atypical Antipsychotics in the Treatment of Schizophrenia and Depression. *Int. J. Mol. Sci.* 23 10624. 10.3390/ijms231810624 36142523 PMC9500595

[B35] Ramírez M. (2013). Multiple organ dysfunction syndrome. *Curr. Probl. Pediatr. Adolesc. Health Care* 43 273–277. 10.1016/j.cppeds.2013.10.003 24295608

[B36] Rosário-Ferreira N. Baptista S. J. Barreto C. A. V. Rodrigues F. E. P. Silva T. F. D. Ferreira S. G. F. (2021). In silico end-to-end protein-ligand interaction characterization pipeline: The case of SARS-CoV-2. *ACS Synth. Biol.* 10 3209–3235. 10.1021/acssynbio.1c00368 34736321

[B37] Shen S. Zhang Y. Yin Z. Zhu Q. Zhang J. Wang T. (2022). Antiviral activity and mechanism of the antifungal drug, anidulafungin, suggesting its potential to promote treatment of viral diseases. *BMC Med.* 20:359. 10.1186/s12916-022-02558-z 36266654 PMC9585728

[B38] Smolders E. J. Jansen A. M. E. Ter Horst P. G. J. Rockstroh J. Back D. J. Burger D. M. (2019). Viral hepatitis C therapy: Pharmacokinetic and pharmacodynamic considerations: A 2019 update. *Clin. Pharmacokinet.* 58 1237–1263. 10.1007/s40262-019-00774-0 31114957 PMC6768915

[B39] Suhandi C. Wilar G. Narsa A. C. Mohammed A. F. A. El-Rayyes A. Muchtaridi M. (2024). Updating the pharmacological effects of α-mangostin compound and unraveling its mechanism of action: A computational study review. *Drug Des. Devel. Ther.* 18 4723–4748. 10.2147/dddt.S478388 39469723 PMC11514645

[B40] Sun Y. Li J. Gao G. F. Tien P. Liu W. (2018). Bunyavirales ribonucleoproteins: The viral replication and transcription machinery. *Crit. Rev. Microbiol.* 44 522–540. 10.1080/1040841x.2018.1446901 29516765

[B41] Takahashi T. Maeda K. Suzuki T. Ishido A. Shigeoka T. Tominaga T. (2014). The first identification and retrospective study of Severe Fever with Thrombocytopenia Syndrome in Japan. *J. Infect. Dis.* 209 816–827. 10.1093/infdis/jit603 24231186 PMC7107388

[B42] Tani H. Fukuma A. Fukushi S. Taniguchi S. Yoshikawa T. Iwata-Yoshikawa N. (2016). Efficacy of T-705 (Favipiravir) in the treatment of infections with lethal severe fever with thrombocytopenia syndrome virus. *mSphere* 1:e00061-15. 10.1128/mSphere.00061-15. 27303697 PMC4863605

[B43] Townsley D. M. Scheinberg P. Winkler T. Desmond R. Dumitriu B. Rios O. (2017). Eltrombopag added to standard immunosuppression for aplastic anemia. *N. Engl. J. Med.* 376 1540–1550. 10.1056/NEJMoa1613878 28423296 PMC5548296

[B44] Trott O. Olson A. J. (2010). AutoDock Vina: Improving the speed and accuracy of docking with a new scoring function, efficient optimization, and multithreading. *J. Comput. Chem.* 31 455–461. 10.1002/jcc.21334 19499576 PMC3041641

[B45] Wang P. Liu L. Liu A. Yan L. He Y. Shen S. (2020). Structure of severe fever with thrombocytopenia syndrome virus L protein elucidates the mechanisms of viral transcription initiation. *Nat. Microbiol.* 5 864–871. 10.1038/s41564-020-0712-2 32341479

[B46] Wang Y. Lin H. Q. Wang P. Hu J. S. Ip T. M. Yang L. M. (2017). Discovery of a Novel HIV-1 Integrase/p75 interacting inhibitor by docking screening, biochemical assay, and in vitro studies. *J. Chem. Inf. Model.* 57 2336–2343. 10.1021/acs.jcim.7b00402 28837332

[B47] Wu J. Power H. Miranda-Saksena M. Valtchev P. Schindeler A. Cunningham A. L. (2022). Identifying HSV-1 inhibitors from natural compounds via virtual screening targeting surface glycoprotein D. *Pharmaceuticals* 15:361. 10.3390/ph15030361 35337158 PMC8955139

[B48] Wurzel R. Ray P. Major-Walker K. Shannon J. Rittmaster R. (2007). The effect of dutasteride on intraprostatic dihydrotestosterone concentrations in men with benign prostatic hyperplasia. *Prostate Cancer Prostatic Dis.* 10 149–154. 10.1038/sj.pcan.4500931 17189955

[B49] Yu X. J. Liang M. F. Zhang S. Y. Liu Y. Li J. D. Sun Y. L. (2011). Fever with thrombocytopenia associated with a novel bunyavirus in China. *N. Engl. J. Med.* 364 1523–1532. 10.1056/NEJMoa1010095 21410387 PMC3113718

[B50] Zhan J. Wang Q. Cheng J. Hu B. Li J. Zhan F. (2017). Current status of severe fever with thrombocytopenia syndrome in China. *Virol. Sin.* 32 51–62. 10.1007/s12250-016-3931-1 28251515 PMC6598917

[B51] Zhang X. Liu Y. Zhao L. Li B. Yu H. Wen H. (2013). An emerging hemorrhagic fever in China caused by a novel bunyavirus SFTSV. *Sci. China Life Sci.* 56 697–700. 10.1007/s11427-013-4518-9 23917841

[B52] Zheng X. Zhang Y. Zhang L. Yang T. Zhang F. Wang X. (2024). Taurolithocholic acid protects against viral haemorrhagic fever via inhibition of ferroptosis. *Nat. Microbiol.* 9 2583–2599. 10.1038/s41564-024-01801-y 39294459

[B53] Zhou H. Sun Y. Wang Y. Liu M. Liu C. Wang W. (2013). The nucleoprotein of severe fever with thrombocytopenia syndrome virus processes a stable hexameric ring to facilitate RNA encapsidation. *Protein Cell* 4 445–455. 10.1007/s13238-013-3901-4 23702688 PMC4875558

[B54] Zhuang L. Sun Y. Cui X. M. Tang F. Hu J. G. Wang L. Y. (2018). Transmission of severe fever with thrombocytopenia syndrome virus by haemaphysalis longicornis ticks, China. *Emerg. Infect. Dis.* 24 868–871. 10.3201/eid2405.151435 29664718 PMC5938789

