# Supplementary material for: Identification of lurasidone as a potent inhibitor of severe fever with thrombocytopenia syndrome virus by targeting the viral nucleoprotein
Source: Front Microbiol. 2025 Apr 28;16:1578844. doi: 10.3389/fmicb.2025.1578844 (PMC12066758; doi:10.3389/fmicb.2025.1578844)
Supplement: Supplementary file 2 [file Data_Sheet_1.pdf]

Supplementary information

Identification of Lurasidone as a Potent Inhibitor of Severe Fever with Thrombocytopenia Syndrome Virus by Targeting the Viral Nucleoprotein

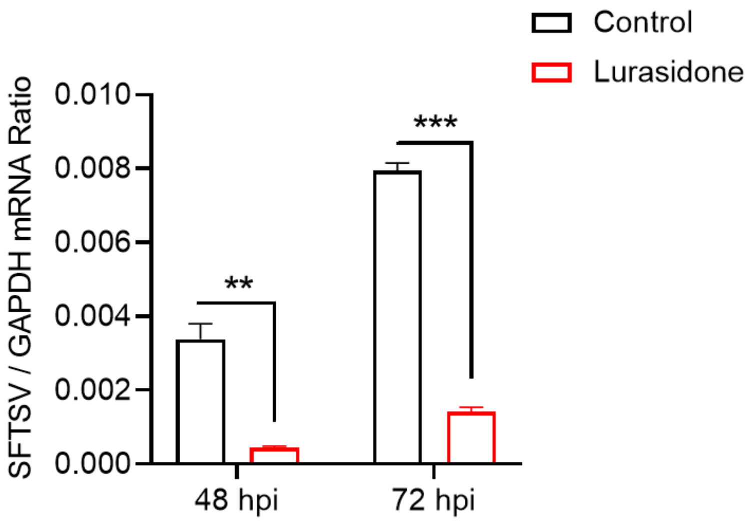

Supplementary Figure 1. Lurasidone Inhibits SFTSV Replication. Vero cells were infected with SFTSV (MOI = 0.1) in the presence of lurasidone (10 μM) or DMSO as a control. Infected cells were collected at 48 and 72 hpi, and viral genome levels were quantified by qRT-PCR.

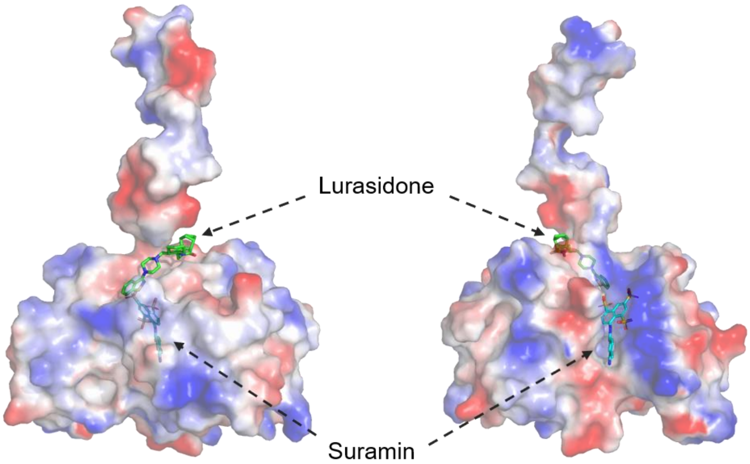

Supplementary Figure 2. Predicted Binding Sites of Lurasidone and Suramin within SFTSV-NP. The binding sites of lurasidone and suramin within the RNA binding cavity of SFTSV-NP were predicted using AutoDock Vina.

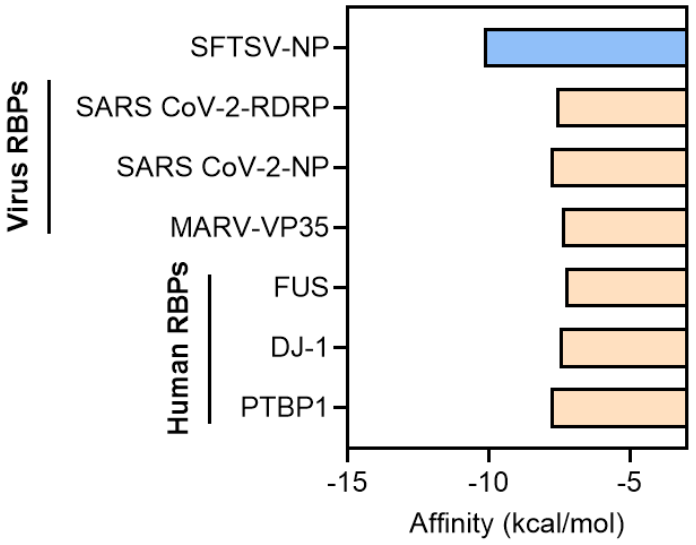

Supplementary Figure 3. ΔG Calculation for NP Docked with RBPs. ΔG values for NP docked with viral and human RBPs were calculated using AutoDock Vina. Virus RBPs: SARS-COV-2-RDRP (PDB ID: 7BTF), SARS-COV-2-NP (PDB ID: 6WKP), MARV-VP35 (Marburg virus Polymerase cofactor VP35, PDB ID: 4GH9); Human RBPs: FUS (PDB ID: 2LCW), DJ-1 (Human DJ-1 protein, PDB ID: 1J42), PTBP1 (Polypyrimidine tract-binding protein 1, PDB ID: 1SJQ).
